# Risk Factors for Postpartum Depressive Symptoms in Japan: A Longitudinal Study From the Second Trimester to Three Months Postpartum

**DOI:** 10.7759/cureus.98864

**Published:** 2025-12-10

**Authors:** Hitomi Kanekasu, Hiroko Watanabe, Satomi Hara, Hitomi Ando

**Affiliations:** 1 Graduate School of Nursing and Social Welfare Sciences, Fukui Prefectural University, Fukui, JPN; 2 Department of Children and Women's Health, The University of Osaka Graduate School of Medicine, Osaka, JPN; 3 College of Nursing Art and Science, University of Hyogo, Akashi, JPN

**Keywords:** depressive symptoms, perinatal, postnatal, prevalence, risk factor

## Abstract

Background

Postnatal depressive symptoms have been reported to have a negative impact on infant development and mother-child interactions. Therefore, it is necessary to prevent postpartum depression during pregnancy and in the early postpartum period. This study aimed to investigate the risk factors for postpartum depressive symptoms up to three months postpartum.

Methods

Data were collected from self-reported questionnaires and participants’ medical records. We recruited women in their second trimester of pregnancy, and 90 participants completed the questionnaires.

Results

Multivariate logistic regression analysis showed that weight gain during pregnancy was related to postpartum depressive symptoms at two to five days postpartum. Childbirth satisfaction and one-minute Apgar scores were associated with depressive symptoms at two weeks postpartum. Childbirth satisfaction and bonding failure at one month postpartum were related to postpartum depressive symptoms at one month postpartum.

Conclusions

We consider the factors identified in this study to be useful indicators for assessing postpartum depressive symptoms. Screening for postpartum depressive symptoms during each period could be beneficial as the risk factors differed between periods.

## Introduction

Perinatal depression is a depressive symptom that occurs during pregnancy or within the first year postpartum [1]. In a recent Japanese meta-analysis, the prevalence of depressive symptoms was calculated as 14.0% in the second trimester, 16.3% in the third trimester, 15.1% within one month postpartum, 11.6% from one to three months postpartum, and 11.5% from three to six months postpartum [2]. The prevalence of depressive symptoms appears to increase as childbirth approaches and decreases during the postpartum period.

Perinatal depressive symptoms are a significant mental health concern. In particular, postnatal depressive symptoms have been reported to have a negative impact on infant development and mother-child interactions [1]. It has also been suggested that there is an association between maternal suicide and mental health within one year postpartum. In Japan, 60% of women who committed suicide postpartum were diagnosed with mental disorders, such as postpartum depression [3]. Moreover, the highest number of suicides occurred four months postpartum [3]. Therefore, it is necessary to prevent postpartum depression during pregnancy and in the early postpartum period.

Many risk factors for postnatal depression have been identified in previous studies. According to an umbrella review, the most common risk factors were high life stress, lack of social support, current or past abuse, prenatal depression, and marital or partner dissatisfaction [4]. In addition, a recent systematic review from the Middle East reported poor economic status, pregnancy-associated complications, low education, unplanned pregnancy, being a housewife, inadequate social support from family members, and feeding by formula as risk factors [5]. Most of these are maternal socio-demographic or prenatal factors; there are a few intrapartum or postpartum factors as well. In the past several years, associations between postpartum depression and mode of delivery [6], preterm birth [7], childbirth satisfaction [8], and bonding failure [9] have also been reported; studies that include these risk factors in addition to common risk factors are needed. A narrative review reported that various biological, psychological, and social factors are associated with postpartum depression [10]. Furthermore, the risk factors for postpartum depressive symptoms may vary over time because the prevalence of postpartum depressive symptoms changes throughout the perinatal period. Therefore, it is necessary to clarify the risk factors for each postpartum period.

This study aimed to investigate the risk factors for postpartum depressive symptoms during each postpartum period. We examined risk factors at two to five days, two weeks, one month, and three months postpartum because the prevalence of depressive symptoms until around three months postpartum is generally high.

## Materials and methods

Participants and procedure

The current study is a longitudinal observational study. We recruited women in their second trimester of pregnancy at a hospital in Kyoto, Japan, from April 2018 to September 2019. The inclusion criteria were (a) primipara, (b) singleton pregnancy, (c) 20-40 years old, and (d) Japanese. The exclusion criteria were (a) having chronic diseases such as essential hypertension and diabetes types 1 and 2, (b) mental diseases such as schizophrenia and epilepsy, and (c) fetal disorders.

Participants completed self-reported questionnaires at five time periods: second trimester, third trimester, two to five days postpartum, one month postpartum, and three months postpartum. The questionnaires consisted of maternal demographic information, depressive symptoms, childbirth satisfaction, and mother-infant bonding, among others. Details of the questionnaires used in the present research are included in the Appendix. Perinatal medical information and depressive symptoms at two weeks and one month postpartum were obtained from the participants’ medical records.

Measures and instruments

Participants’ Characteristics

Maternal demographic information included maternal age, education, marital status, marital quality, social support, and so on. Marital quality was measured using the Quality of Marriage Index (QMI), which comprises six items rated on a four-point Likert scale. The total QMI score, which ranges from 6 to 24, is used as the marital quality score. Higher scores indicate better marital quality. The QMI has been used in previous studies of postpartum women [11]. We used the Japanese version of QMI developed by Moroi [12]. High validity and reliability of the Japanese version of the QMI have been reported [12]. In the present study, Cronbach’s α for the QMI was 0.899. Data on prenatal social support were collected in the third trimester, and postnatal social support was collected one month postpartum. Prenatal and postpartum social support was measured using the Multidimensional Scale of Perceived Social Support (MSPSS), comprising 12 items rated on a seven-point Likert scale. The mean for the MSPSS score, ranging from 1 to 7, was used for the social support score. Higher scores indicate better social support. The MSPSS is widely used in studies of postpartum women [13]. We used the Japanese version of the MSPSS developed by Iwasa et al. [14], which is reported to have high validity and reliability. In the present study, Cronbach’s α for the MSPSS was 0.917 in the third trimester and 0.931 at one month postpartum. The Japanese version of these two scales has been published in book form and is authorized for use in research.

The perinatal medical information included history of miscarriage, fertility treatment, pre-pregnancy body mass index (BMI), weight gain during pregnancy, hemoglobin in the third trimester, mode of delivery, gestational age, infant birth weight, Apgar score, and duration of hospitalization. Pre-pregnancy BMI and weight gain during pregnancy were based on the criteria of the Ministry of Health, Labor, and Welfare and the Japan Society for the Study of Obesity. We also obtained information on the diagnosis of hypertension disorders of pregnancy (HPD), gestational diabetes mellitus (GDM), threatened premature delivery, and premature rupture of membranes. HPD and GDM were diagnosed according to the Japan Society of Obstetrics and Gynecology criteria. In this study, anemia during pregnancy was defined as hemoglobin <11.0 g/dL, preterm birth was defined as birth before 37-week gestational age, and low birth weight was defined as birth weight <2,500 g at birth. Pregnancy complications included HPD, GDM, and threatened premature deliveries.

Depressive Symptoms

Depressive symptoms were assessed using the Edinburgh Postnatal Depression Scale (EPDS) developed by Cox et al. [15]. The EPDS comprises 10 items, ranging from 0 to 3, with the total score ranging from 0 to 30. The Japanese version of the EPDS, using a cutoff point of 8/9, reported that the sensitivity and specificity were 75% and 93%, respectively [16]. This cutoff point has been commonly used in Japanese prenatal and postnatal studies [2]. In this study, Cronbach’s α for the EPDS was 0.710, 0.790, 0.875, 0.777, 0.774, and 0.653 in the second trimester, third trimester, two to five days postpartum, two weeks postpartum, one month postpartum, and three months postpartum, respectively. The Japanese version of EPDS has been published in book form and is officially authorized for research use. Permission for the EPDS was also obtained from the British Journal of Psychiatry.

Childbirth Satisfaction

Childbirth satisfaction was measured using the visual analog scale (VAS). The VAS is a 100 mm horizontal line that has been used to measure a variety of subjective phenomena [17]. In previous studies, the VAS has been used to measure the levels of patients’ satisfaction [18]. In addition, VAS has been used in some studies that investigated childbirth satisfaction [19]. Participants were required to mark a spot on the line corresponding to their childbirth satisfaction on a scale of 0-100 mm. Higher scores indicate higher childbirth satisfaction.

Bonding Failure

Bonding failure was measured using the Japanese version of the Mother-to-Infant Bonding Scale (MIBS), developed by Yoshida et al. [20]. The MIBS consists of 10 items rated on a four-point Likert scale, ranging from 0 to 3. Higher scores indicate worse mother-to-infant bonding. Yoshida et al. [20] reported the acceptable validity and reliability of the Japanese version of the MIBS. In the present study, Cronbach’s α for the MIBS was 0.791 at one month postpartum and 0.853 at three months postpartum. The Japanese version of MIBS has been formally published and is available for academic research use.

Statistical analysis

Normal distributions of continuous variables were validated using the Shapiro-Wilk test. Fisher's exact test was used to compare depressive symptoms in each period.

Bivariate logistic regression analysis was used to examine the relationship between participant characteristics and postpartum depressive symptoms. The participant characteristics variables as risk factors were obtained from previous studies [4,5,8]. Several variables could not be included in the analysis due to extreme skewness in the distribution of variables, such as marital status, unplanned pregnancy, and preterm infants. These variables caused separation in the regression analysis.

Multivariate logistic regression analyses for depressive symptoms at two to five days, two weeks, and one month were conducted. Variables with a significance probability of less than 0.1 in a bivariate logistic regression analysis were used as independent variables using the forced entry method. Multicollinearity was examined using Spearman's rank correlation coefficient. There was a moderate correlation between social support in the third trimester and at one month postpartum (p = 0.000, r^s^ = 0.653) and social support at one month postpartum and bonding failure at one month postpartum (p = 0.000, r^s^ = -0.477); therefore, social support at one month postpartum was not included in the model at one month postpartum. We could not analyse depressive symptoms at three months postpartum because there was only one variable with a significance probability of less than 0.1.

Statistical significance was defined as *p* < 0.05. All statistical analyses were performed using SPSS Statistics for Windows, version 27.0 (IBM Japan, Tokyo, Japan).

Ethical approval

This study was reviewed and approved by the Osaka University Research Ethics Committee (16464-2). All procedures performed in this study involving human participants were in accordance with the ethical standards of the institutional or national research committee and with the 1964 Helsinki Declaration and its later amendments or comparable ethical standards.

## Results

Participant characteristics

One hundred thirty-five primiparas participated in this study. Fourteen participants were transferred to other hospitals during pregnancy. Further, 38 participants did not complete one or more questionnaires. Finally, 90 participants were included in the analysis at two to five days, two weeks postpartum, and one month postpartum. Furthermore, 83 participants were included in the analysis at three months postpartum.

Table 1 summarizes the participants’ characteristics. The mean maternal age was 32.0 ± 3.7 years, and 49 (54.4%) were university graduates. In total, 66 (73.3%) of the infants were born by vaginal delivery, and 50 (57.8%) were men. In addition, three infants (3.3%) were born prematurely, and four (4.4%) had low birth weights.

**Table 1:** Participant characteristics Abbreviation: BMI = Body mass index, HDP = Hypertensive disorders of pregnancy, Hgb = Hemoglobin, GDM = Gestational diabetes mellitus, MSPSS = Multidimensional Scale of Perceived Social Support, QMI = Quality Marriage Index, VAS = Visual analog scale †Based on the criteria of the Ministry of Health, Labor and Welfare, and the criteria of the Japan Society for the study of obesity

n = 90		N (%) / Mean ± SD/ Median (IQR)
Age (years)		32.0 ± 3.7
Education	Secondary or High school	9 (10.0)
	Junior or Technical College	25 (27.8)
	University	49 (54.4)
	Graduate school	7 (7.8)
Marital status	Married	87 (96.7)
	Single	3 (3.3)
Marital quality (QMI)		21.5 (19.0, 24.0)
Family form	Nuclear family	87 (96.7)
	Extended family	3 (3.3)
Household income/year (JPY)	< 2 million	3 (3.3)
	2-5.9 million	52 (57.8)
	≥ 6 million	35 (38.9)
Employed during pregnancy	Yes	57 (63.3)
Social support in the third trimester (MSPSS)		5.96 (5.56, 6.42)
History of miscarriage	Yes	18 (20.0)
Infertility treatment	Yes	22 (24.4)
Unplanned pregnancy	Yes	7 (7.8)
Smoking during pregnancy	Yes	0 (0)
Alcohol during pregnancy	Yes	1 (1.1)
Pre-pregnancy BMI ^†^	Underweight (<18.5)	11 (12.2)
	Normal weight (18.5-24.5)	73 (81.1)
	Overweight (25.0-29.9)	6 (6.7)
Weight gain during pregnancy^ †^	Below	15 (16.7)
	Within	40 (44.4)
	Above	35 (38.9)
Anemia in the third trimester (Hgb < 11.0g/dL)	Yes	32 (35.6)
HPD	Yes	5 (5.6)
GDM	Yes	2 (2.2)
Threatened premature delivery	Yes	4 (4.4)
Premature rupture of the membrane	Yes	18 (20.0)
Mode of delivery	Vaginal delivery	66 (73.3)
	C-section	24 (26.6)
Support person during birth	Yes	82 (91.1)
Childbirth satisfaction (VAS)		8.5 (7.0, 9.8)
Infant sex	Male	50 (57.8)
	Female	38 (42.2)
Gestational age (week)		39.9 (38.9, 40.4)
Infant birth weight (g)		3089.4 ± 331.7
Preterm infant (< 37 weeks)	Yes	3 (3.3)
Low birth weight (< 2500 g)	Yes	4 (4.4)
1-minute Apgar score		9.0 (8.0, 9.0)
5-minute Apgar score		9.0 (9.0, 10.0)
Incubator management	Yes	5 (5.6)
Duration of hospitalization (days)		4.0 (4.0, 5.0)
Feeding status at discharge	Only breast milk	3 (3.3)
	Formula or mixed	87 (96.7)
Social support at 1 month postpartum (MSPSS)		6.20 (5.80, 6.80)

Prevalence of maternal depressive symptoms

Depression symptoms (EPDS ≥9) were 14 (15.6%), 14 (15.6%), 26 (28.9%), 14 (15.6%), 8 (8.9%), and 4 (4.8%) in the second trimester, third trimester, two-five days postpartum, two weeks postpartum, one month postpartum, and three months postpartum, respectively. Fisher's exact test showed that depressive symptoms in the second trimester were related to depressive symptoms in the third trimester (p = 0.034) and depressive symptoms at two weeks postpartum (p = 0.035). Depressive symptoms in the second trimester were not associated with depressive symptoms at two to five days, one month, and three months postpartum (p = 0.375, p = 0.105, p = 0.501).

Bivariate analysis between independent variables and postpartum depressive symptoms

The results of the bivariate logistic regression analysis are presented in Table 2. At two to five days postpartum, depression symptoms in the third trimester, weight gain during pregnancy, and childbirth satisfaction were associated with postpartum depression symptoms. At two weeks postpartum, depressive symptoms in the third trimester and childbirth satisfaction were associated with postpartum depressive symptoms. At one month postpartum, prenatal social support, childbirth satisfaction, and bonding failure at one month postpartum were associated with postpartum depressive symptoms. Bonding failure at three months postpartum was related to postpartum depressive symptoms.

**Table 2:** Bivariate analysis between independent variables and postpartum depressive symptoms Abbreviation: BMI = Body mass index, MIBS = Mother-to-Infant Bonding Scale, MSPSS = Multidimensional Scale of Perceived Social Support, QMI = Quality Marriage Index. † Pregnancy complications include hypertensive disorders of pregnancy, gestational diabetes mellitus, and threatened premature delivery. Bivariate logistic regression analysis. +p ≤ 0.1, *p < 0.05, **p <0.01, ***p < 0.001

Variables	Two to five days postpartum (n=90)	Two weeks postpartum (n=90)	One month postpartum (n=90)	Three months Postpartum (n=83)
	OR (95% CI)	OR (95% CI)	OR (95% CI)	OR (95% CI)
Age (years)	1.05 (0.93-1.19)	1.08 (0.92-1.27)	1.02 (0.84-1.24)	1.13 (0.85-1.50)
Pre-pregnancy BMI	0.71 (0.81-1.19)	0.920 (0.71-1.21)	0.99 (0.73-1.34)	0.73 (0.41-1.30)
Education				
Secondary or High school	Reference	Reference	Reference	-
Junior or Technical college	1.97 (0.34-11.57)	0.67 (0.10-4.46)	0.30 (0.04-1.75)	-
University or Graduate school	1.28 (0.24-6.87)	0.58 (0.10-3.33)	0.17 (0.04-1.75)	-
Marital quality (QMI)	1.02 (0.89-1.18)	0.97 (0.82-1.15)	0.91 (0.75-1.10)	0.92 (0.71-1.20)
Household income/year (JPY)				
< 6 million	Reference	Reference	-	-
≥ 6 million	0.47 (0.18-1.28)	0.38 (0.10-1.45)	-	-
Employed during pregnancy				
No	Reference	Reference	Reference	Reference
Yes	0.71 (0.28-1.82)	1.05 (0.32-3.45)	0.55 (0.13-2.35)	0.58 (0.08-4.34)
Social support in the third trimester (MSPSS)	0.55 (0.29-1.03) +	0.51 (0.23-1.12) +	0.31 (0.11-0.85) *	0.81 (0.21-3.16)
History of miscarriage				
No	Reference	Reference	Reference	Reference
Yes	0.93 (0.30-2.45)	1.11 (0.28-4.48)	2.68 (0.58-12.46)	1.22 (0.12-12.44)
Infertility treatment				
No	Reference	Reference	Reference	Reference
Yes	0.66 (0.21-2.02)	1.29 (0.63-4.61)	1.03 (0.19-5.53)	0.98 (0.10-9.99)
Depressive symptoms in the second trimester				
No	Reference	Reference	Reference	Reference
Yes	1.46 (0.44-4.85)	1.61 (0.39-6.72)	3.87 (0.81-18.54) +	1.86 (0.18-19.42)
Depressive symptoms in the third trimester				
No	Reference	Reference	Reference	Reference
Yes	4.30 (1.32-14.03) *	4.14 (1.13-15.11) *	1.94 (0.35-10.79)	5.58 (0.72-43.55)
Anemia in the third trimester				
No	Reference	Reference	Reference	Reference
Yes	0.84 (0.33-2.15)	0.99 (0.30-3.26)	0.912 (0.20-4.01)	0.55 (0.07-4.11)
Weight gain during pregnancy (kg)	1.17 (1.01-1.34) *	1.12 (0.95-1.33)	0.98 (0.80-1.19)	0.94 (0.72-1.22)
Pregnancy complications				
No	Reference	Reference	Reference	-
Yes	0.51 (0.10-2.54)	0.51 (0.06-4.32)	1.03(0.11-9.26)	-
Premature rupture of the membrane				
No	Reference	Reference	Reference	Reference
Yes	2.10 (0.82-7.01)	1.11 (0.28-4.48)	2.68 (0.58-12.46)	1.31 (0.13-13.47)
Mode of delivery				
Vaginal delivery	Reference	Reference	Reference	Reference
C-section	1.33 (0.49-3.65)	1.12 (0.32-3.98)	3.10 (0.71-13.55)	0.98 (0.097-9.999)
Support person during birth	3.01 (0.36-26.29)	1.12 (0.15-11.64)	-	-
Childbirth satisfaction	0.74 (0.59-0.94) *	0.69 (0.53-0.90) **	0.66 (0.48-0.90) *	0.77 (0.51-1.17)
Gestational age (week)	1.01 (0.95-1.07)	1.05 (0.97-1.14)	1.06 (0.95-1.17)	0.97 (0.86-1.09)
Infant birth weight (g)	1.00 (0.998-1.001)	1.00 (0.998-1.002)	0.999 (0.997-1.002)	1.00 (0.998-1.004)
1-minute Apgar score	1.36 (0.71-2.64)	0.56 (0.61-1.03) +	1.32 (0.44-3.92)	6.45 (0.45-93.51)
5-minute Apgar score	1.47 (0.67-3.20)	0.64 (0.28-1.45)	0.75 (0.27-2.09)	-
Incubator management				
No	Reference	Reference	Reference	-
Yes	1.67 (0.18-15.67)	0.72 (0.08-6.99)	0.36 (0.04-3.67)	-
Duration of hospitalization	0.90 (0.51-1.59)	1.45 (0.81-2.60)	1.24 (0.59-2.63)	1.21 (0.45-3.30)
Social support at one month postpartum (MSPSS)	-	-	0.42 (0.17-1.05) +	1.30 (0.29-5.81)
Bonding failure at one month postpartum (MIBS)	-	-	1.42 (1.13-1.78) **	1.23 (0.97-1.56) +
Bonding failure at three months postpartum (MIBS)	-	-	-	1.36 (1.06-17.5) *

Multivariate analysis of independent variables and postpartum depressive symptoms

The results of the multivariate logistic regression analysis are presented in Table 3. At two to five days postpartum, only weight gain during pregnancy was associated with postpartum depressive symptoms. At two weeks postpartum, childbirth satisfaction and 1-minute Apgar scores were associated with postpartum depressive symptoms. At one month postpartum, childbirth satisfaction and bonding failure at one month postpartum were related to postpartum depression symptoms.

**Table 3:** Multivariate analysis between independent variables and postpartum depressive symptoms Abbreviation: MIBS = Mother-to-Infant Bonding Scale, MSPSS = Multidimensional Scale of Perceived Social Support, VAS = Visual Analog Scale. Multivariate logistic regression analysis, Forced Entry method, * p < 0.05, ** p <0.01, ***p < 0.001.

Parameters	Two to five days postpartum	Two weeks postpartum	One month postpartum
	OR (95% CI)	OR (95% CI)	OR (95% CI)
Social support in the third trimester (MSPSS)	0.64 (0.31-1.35)	0.66 (0.29-1.51)	0.37 (0.07-1.88)
Weight gain during pregnancy (kg)	1.17 (1.003-1.37) *	-	-
Depressive symptoms in the second trimester	-	-	7.83 (0.48-126.45)
Depressive symptoms in the third trimester	2.30 (0.65-8.90)	2.87 (0.70-11.73)	-
Childbirth satisfaction (VAS)	0.77 (0.60-1.00)	0.75 (0.56-0.99) *	0.52 (0.29-0.92) *
1-minute Apgar score	-	0.48 (0.25-0.83) *	-
Bonding failure at one month postpartum (MIBS)	-	-	1.59 (1.12-2.25) **
Omnibus tests of model coefficients	χ2 (df = 4) = 16.098	χ2 (df = 4) = 15.386	χ2 (df = 4) = 23.085
	p = 0.003	p = 0.004	p = 0.000
Homer- Lemeshow test	χ2 (df = 8) = 7.380	χ2 (df = 8) = 9.962	χ2 (df = 8) = 7.275
	p = 0.496	p = 0.268	p = 0.507

## Discussion

In our study, the prevalence of depressive symptoms was the highest at 28.9% at two to five days postpartum. The prevalence in the second trimester, third trimester, and two weeks postpartum was 15.6%. The prevalence at one month and three months postpartum was less than 9%, and it decreased over time. The prevalence of depressive symptoms appears to increase as delivery approaches, peaking in the immediate postpartum period and decreasing in the postpartum period. The peak postnatal prevalence in the immediate postpartum period was similar to that reported by Falah-Hassani et al. [21], who reported that the prevalence of depressive symptoms was 29.6% at one week, 23.0% at four weeks, and 20.4% at eight weeks postpartum. Rapid postpartum hormonal changes may contribute to depressive symptoms [22]. In our study, the prevalence of prenatal depressive symptoms was similar; however, postpartum depressive symptoms at one month and three months postpartum were slightly lower than those in a previous Japanese meta-analysis [2]. In our research hospital, routine screenings of depressive symptoms using the EPDS were conducted at two weeks and one month postpartum. Therefore, the care provider could conduct mental healthcare for women with depressive symptoms, which might have affected the prevalence.

The results of the multivariable logistic regression showed that weight gain during pregnancy was related to depressive symptoms at two to five days postpartum, and childbirth satisfaction and one-minute Apgar scores were associated with postpartum depressive symptoms at two weeks postpartum. In addition, childbirth satisfaction and bonding failure at one month postpartum were associated with depressive symptoms at one month postpartum. In other words, our study revealed that the risk factors for depressive symptoms differ depending on the postpartum period. Screening for the presence or absence of each factor at each stage is considered effective in preventing postpartum depressive symptoms.

A few studies have investigated an association between weight gain during pregnancy or Apgar score and postpartum depressive symptoms; there were no relationships among weight gain during pregnancy, Apgar scores, and postpartum depressive symptoms [23]. However, there was an association between weight gain during pregnancy and postpartum depressive symptoms in the present study. This result may be related to the body image of Japanese women, as they tend to have a strong desire to be thin [24]; therefore, weight gain during pregnancy may be associated with postpartum depressive symptoms. Furthermore, it has been reported that body image dissatisfaction is consistently but weakly associated with the onset of prenatal and postpartum depressive symptoms [25]. Greater weight gain during pregnancy may reduce women’s body image satisfaction and affect their depressive symptoms.

Childbirth satisfaction is related to postpartum depressive symptoms at both two weeks and one month postpartum. It was also associated with depressive symptoms at two to five days postpartum when bivariate results were included. Childbirth satisfaction is suggested to be a strong predictive risk factor for long-term postpartum depressive symptoms up to one month postpartum. Several studies have also reported this association [8,26].

Bonding failure at one month postpartum was associated with depressive symptoms at one month postpartum. In addition, bonding failure at three months postpartum was related to postpartum depressive symptoms at three months postpartum when bivariate results were included. Kasamatsu et al. [9] reported that depressive symptoms were associated with bonding failure at one month and six months postpartum. In addition, Tsuchida et al. [27] reported a relationship between bonding failure and postpartum depressive symptoms at one month postpartum. Bonding failure appears to be a risk factor that strongly influences depressive symptoms, as many studies have reported. However, it may be difficult to use this as a predictor because of the bonding failure associated with depressive symptoms in the same period. Bivariate analysis showed that depressive symptoms in the third trimester were related to two to five days and two weeks postpartum. Similarly, depressive symptoms during pregnancy were associated with postpartum depressive symptoms in a bivariate analysis; however, they were not associated with a multivariate analysis in a previous study [28]. Many studies using multivariate analysis have reported that prenatal depressive symptoms are associated with postpartum depressive symptoms [4]. The discrepancies in these results might be attributed to the differences in the survey period, survey participants, etc. In our study, the limited number of women with postpartum depressive symptoms may have contributed to the lack of an association. This needs to be investigated with a larger number of participants, including women with depressive symptoms.

Social support in the third trimester was related to depressive symptoms at one month postpartum in the bivariate analysis but not in the multivariate analysis. Many studies have reported a relationship between social support and depressive symptoms [4,29]. However, an Indonesian study reported that perceptions of postpartum support were not associated with postpartum depressive symptoms. Nurbaeti et al. [30] speculated that the culture of receiving support in housework and childcare from women’s mothers might have influenced their results. Japan has a similar culture where many women are supported by their mothers in childcare. In fact, the postnatal MSPSS mean score was as high as 6.20 out of 7 points in the present study, though our study did not measure whether participants received help from their mothers. In addition to the MSPSS score, information such as the number or type of social support also needs to be collected.

We investigated the quality of marital relationships; however, marital quality was not associated with postpartum depressive symptoms in our study. Lower marital satisfaction [30], no partner support [29], and intimate partner violence [4,6] were considered risk factors for postpartum depressive symptoms. In our study, more than 95% of study participants were married and had a high QMI score of 21 out of 24. The association between marital quality and postpartum depressive symptoms may not be evident in our study, as most women were satisfied with their marital relationship. However, it is necessary to investigate negative factors, such as current marital problems, partner support, and partner violence.

This study had some limitations. First, the generalizability of the research is limited because only 90 women participated in our study, and the findings are limited to primiparas. Second, several variables, such as marital status, unplanned pregnancy, and preterm infants, could not be included in the analysis because of extreme skewness in the distribution of the variables. In addition, because the number of women with depressive symptoms was marginal, we could not conduct multivariable logistic regression for three months postpartum. We need to investigate this with a larger number of participants, including women with depressive symptoms, unmarried women, and those with unplanned pregnancies or preterm infants. Third, we used only self-reported questionnaires to identify depressive symptoms. Depressive symptoms using EPDS differ from depression as a diagnosis in a strict sense. Finally, we did not investigate prenatal or postnatal mental care of the care providers. If care providers provide mental healthcare to women with depressive symptoms, it is possible that the prevalence or risk factors might be affected. Thus, further surveys are needed to include information on mental care.

## Conclusions

In conclusion, our study identified risk factors for postpartum depressive symptoms at two to five days, two weeks, one month, and three months postpartum among Japanese primiparas. At two to five days postpartum, weight gain during pregnancy was associated with postpartum depressive symptoms; at two weeks postpartum, childbirth satisfaction and one-minute Apgar score were associated with postpartum depressive symptoms; and childbirth satisfaction and bonding failure at one month postpartum were related to depressive symptoms at one month postpartum. We consider these factors to be useful indicators of postpartum depressive symptoms. As the risk factors differ according to the period, we believe that screening at each period contributes to the prevention of postpartum depressive symptoms.

## Appendices

**Table 4:** Details of the questionnaires in present research The EPDS [15,16], MIBS [20], MSPSS [14], and QMI [12] have been published in book form and are officially authorized for research use. Permission for the EPDS was also obtained from the British Journal of Psychiatry. The remaining items were developed by the authors specifically for this study.

Items	Responses
Pregnancy period	
How old were you at the time of this pregnancy? (2nd trimester)	_______ years
Was this pregnancy conceived naturally, or was it the result of fertility treatment? (2nd trimester)	Natural conception, Fertility treatment (Artificial insemination, In vitro fertilization)
Was this a planned pregnancy? (2nd trimester)	Yes, Neither agree nor disagree, No
Do you have any medical conditions that were diagnosed before this pregnancy? (2nd trimester)	None, (please specify) ___________
Which of the following best describes your family structure? (2nd trimester)	Nuclear family, Extended family, Other (please specify): __________
Are you legally married to your partner? (2nd trimester)	Yes, No
What is your highest level of education? (2nd trimester)	Secondary school, High school, Junior or Technical College, University, Graduate school
Are you currently employed? (2nd trimester)	Employed full-time, Employed part-time, Unemployed
What is the approximate annual income of your household? (2nd trimester)	< 2 million, 2 - 5.9 million, ≥ 6 million (JPY)
The Quality of Marriage Index: QMI (2nd trimester)	6 items rated on a 4-point Likert scale
Did you attend any prenatal classes related to childbirth? (3rd trimester)	Yes, No
Do you currently have a habit of smoking? (3rd trimester)	Yes, No
Do you currently have a habit of drinking alcohol? (3rd trimester)	Yes, No
Were you diagnosed with any medical conditions during this pregnancy? (3rd trimester)	Yes, No
Are you currently taking any medications? (3rd trimester)	Yes, No
The Multidimensional Scale of Perceived Social Support: MSPSS (3rd trimester)	12 items rated on a7 -point Likert scale
Postpartum period	
How satisfied were you with your childbirth experience? Please mark an “X” in the box that best matches your feelings, based on your intuition. (2-5 days)	Not satisfied at all ------------ Very satisfied
Was anyone present with you during your delivery? (2-5 days)	No, Yes (husband, mother, other ________)
Multidimensional Scale of Perceived Social Support: MSPSS (1 month)	12 items rated on a7 -point Likert scale
The Edinburgh Postnatal Depression Scale: EPDS (2-5 days, 2weeks, 1 month, 3 months)	10 items rated on a 4-point Likert scale
Mother-to-Infant Bonding Scale: MIBS (1 month, 3 months)	10 items rated on a 4-point Likert scale
